# Forward and Backward Pressure Waveform Morphology in Hypertension

**DOI:** 10.1161/HYPERTENSIONAHA.116.08089

**Published:** 2017-01-11

**Authors:** Ye Li, Haotian Gu, Henry Fok, Jordi Alastruey, Philip Chowienczyk

**Affiliations:** From the British Heart Foundation Centre (Y.L., H.G., H.F., P.C.) and Division of Imaging Sciences and Biomedical Engineering (J.A.), King’s College London, United Kingdom.

**Keywords:** aorta, arterial pressure, dobutamine, nitroglycerin, norepinephrine, phentolamine, pulse wave analysis

## Abstract

We tested the hypothesis that increased pulse wave reflection and altered backward waveform morphology contribute to increased pulse pressure in subjects with higher pulse pressure compared with lower pulse pressure and to actions of vasoactive drugs to increase pulse pressure. We examined the relationship of backward to forward wave morphology in 158 subjects who were evaluated for hypertension (including some normotensive subjects) divided into 3 groups by central pulse pressure: group 1, 33±6.5 mm Hg; group 2, 45±4.1 mm Hg; and group 3, 64±12.9 mm Hg (means±SD) and in healthy normotensive subjects during administration of inotropic and vasomotor drugs. Aortic pressure and flow in the aortic root were estimated by carotid tonometry and Doppler sonography, respectively. Morphology of the backward wave relative to the forward wave was similar in subjects in the lowest and highest tertiles of pulse pressure. Similar results were seen with the inotropic, vasopressor and vasodilator drugs, dobutamine, norepinephrine, and phentolamine, with the backward wave maintaining a constant ratio to the forward wave. However, nitroglycerin, a drug with a specific action to dilate muscular conduit arteries, reduced the amplitude of the backward wave relative to the forward wave from 0.26±0.018 at baseline to 0.19±0.019 during nitroglycerin 30 μg/min IV (*P*<0.01). These results are best explained by an approximately constant amount of reflection of the forward wave from the peripheral vasculature. The amount of reflection can be modified by dilation of peripheral muscular conduit arteries but contributes little to increased pulse pressure in hypertension.

## Abstract 2

Supplemental Digital Content is available in the text.

Central aortic hemodynamics are determined by coupling between the left ventricle and peripheral arterial tree. Pressure waves in the proximal aorta can be separated into a forward wave traveling from the ventricle to the periphery of the arterial tree and a backward wave traveling in the reverse direction.^1,2^ The forward wave is generated primarily by ventricular contraction and has an amplitude that is mainly determined by ventricular contraction and pulse wave velocity (PWV) of the proximal aorta.^3^ The backward wave is thought to be generated by the reflection of the forward wave from downstream segments of the arterial tree and, hence, to be influenced by the characteristics of the peripheral vasculature at the major sites of reflection.^3^ Wave separation, therefore, potentially provides insight into the genesis of pulsatile components of aortic (and peripheral) blood pressure (BP) and, hence, into the increase in pulse pressure that is the major hemodynamic change contributing to hypertension and cardiovascular disease in the aging population.^4^

Most previous studies have used indirect surrogates of the contribution of the backward relative to the forward wave such as augmentation pressure (AP) or have quantified reflection in terms of the ratio of the peak of the forward to backward wave.^5–7^ The aim of this study was to test the hypothesis that increased pulse wave reflection contributes to altered backward waveform morphology and to increased pulse pressure in subjects with higher pulse pressure compared with lower pulse pressure and to actions of vasoactive drugs to increase pulse pressure. We compared forward and backward waveform morphology in subjects with low and high pulse pressure and after changes in pulse pressures induced by a range of inotropic, vasoconstrictor and vasodilator drugs. We used numeric modeling to confirm the interpretation of the experimental results.

## Methods

### Study 1: Central Hemodynamics in Patients With Hypertension

Subjects (n=158, 81 male, aged 46±17 years, mean±SD) were recruited from those who were evaluated for hypertension at Guy’s and St Thomas’ Hypertension Clinic. Although subjects were referred for evaluation of hypertension, BP settled in some subjects and the sample included some who were normotensive; 48% of the subjects were on treatment. Subjects with significant valvular disease, impaired left ventricular systolic function (ejection fraction <45%), and arrhythmias were excluded. The sample included a subsample of 20 subjects in which we have previously published data on hemodynamics (but not with the present focus on waveform morphology).^8^ Anthropometric and clinical data were collected on the day of the research investigations, including height, weight, measurements of systolic and diastolic BP, and the characteristics of hypertensive subjects and are shown in Table. Patients were divided into 3 groups corresponding to tertiles of central pulse pressure (group 1, 33±6.5 mm Hg; group 2, 45±4.1 mm Hg; and group 3, 64±12.9 mm Hg, means±SD) to test the hypothesis that reflection and backward waveform morphology contribute to raised pulse pressure. Hemodynamic measurements were obtained as detailed below.

**Table 1.**
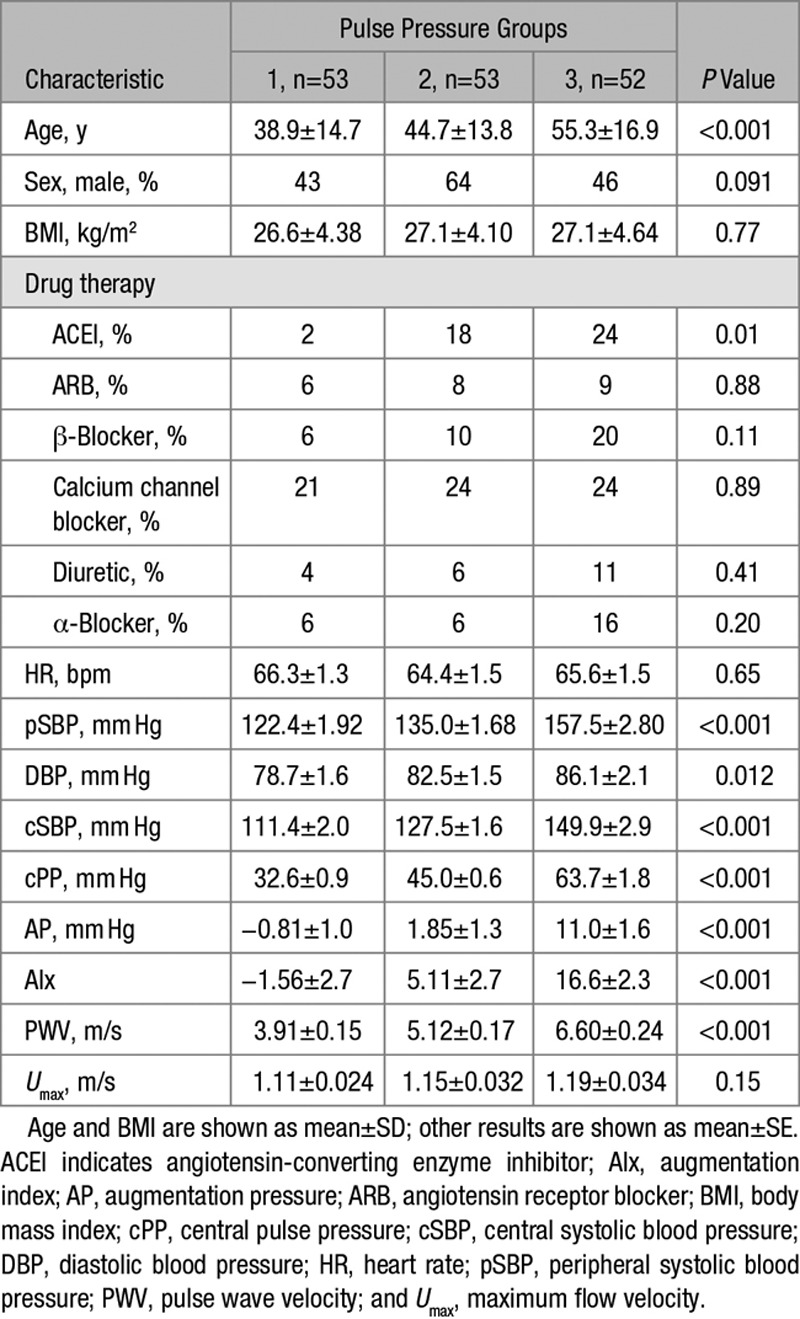
Characteristics of Hypertensive Subjects

### Study 2: Effects of Dobutamine, Norepinephrine, Phentolamine, and Nitroglycerin, on Central Hemodynamics in Normotensive Volunteers

Healthy volunteers (n=13, 10 male, aged 47±10 years, mean±SD) took part in crossover studies to investigate the change in pulsatile hemodynamics during administration of drugs with different inotropic and vasopressor/vasodilator properties: dobutamine (a positive inotrope with some vasodilator actions), norepinephrine (a vasoconstrictor with some inotropic actions), phentolamine (a small artery dilator), and nitroglycerin (predominantly a large artery dilator with some action on ventricular dynamics and also venodilation). Each subject received at least 2 comparator drugs: either the vasopressor agents dobutamine and norepinephrine or the vasodilators phentolamine and nitroglycerin, and data for each drug were obtained on at least 10 subjects. Each drug was given on a different occasion separated by at least 7 days, and the order was randomized. Some hemodynamic data on norepinephrine and dobutamine have previously been published (but not with the present focus on waveform morphology).^8^ Measurements were performed in a quiet temperature controlled (24–26°C) vascular laboratory, and subjects were asked to avoid caffeine and alcohol on the day of the study. On arrival in the vascular laboratory, a peripheral venous catheter was inserted into the left antecubital fossa through which 0.9% saline (Baxter Healthcare) vehicle or drugs dissolved in saline were infused at 1 mL/min using a syringe driver (Injectomat, Agilia; Fresenius Kabi, Bad Homburg vor der Höhe, Germany). After 30-minute resting supine during infusion of saline vehicle, baseline hemodynamic measurements were made as detailed below. On different occasions, dobutamine (2.5, 5, and 7.5 µg/kg per minute; Hameln Pharmaceuticals, Gloucester, United Kingdom), norepinephrine (12.5, 25, and 50 ng/kg per minute; Aguettant, Bristol, United Kingdom), phentolamine (1 mg bolus+25 μg/min, 2 mg+50 μg/min, and 4 mg+100 μg/min; Alliance Pharmaceuticals, Chippenham, United Kingdom), and nitroglycerin (3, 10, and 30 μg/min; Hospira Incorporation, Lake Forest, IL) dissolved in 0.9% saline vehicle were then infused at 1 mL/min, and hemodynamic measurements were repeated at each drug dose when steady state was achieved after at least 7 minutes of infusion. Both studies 1 and 2 were approved by the London Westminster Research Ethics Committee, and written informed consent was obtained.

### Hemodynamic Measurements

Hemodynamic measurements were performed as previously described.^8^ Radial and carotid pressure waveforms were obtained by applanation tonometry performed by an experienced operator using the SphygmoCor system (AtCor, West Ryde, New South Wales, Australia). Approximately 10 cardiac cycles were obtained and ensemble averaged. Waveforms that did not meet the in-built quality control criteria in the SphygmoCor system were rejected. Brachial BP was measured in triplicate by a validated oscillometric method (Omron 705CP; Omron Healthcare, Japan) and used to calibrate radial waveforms and thus to obtain a mean arterial pressure through integration of the radial waveform. Carotid waveforms were calibrated from mean arterial pressure and diastolic brachial BP on the assumption of equality of these pressures at central and peripheral sites.^9^ Ultrasound imaging was performed by an experienced operator using the Vivid-7 ultrasound platform (General Electric Healthcare, Little Chalfont, United Kingdom). Velocity above the aortic valve was recorded using pulsed wave Doppler obtained from an apical 5-chamber view. All ultrasound measurements were averaged over at least 3 cardiac cycles.

### Waveform Postprocessing

**Figure 1.**
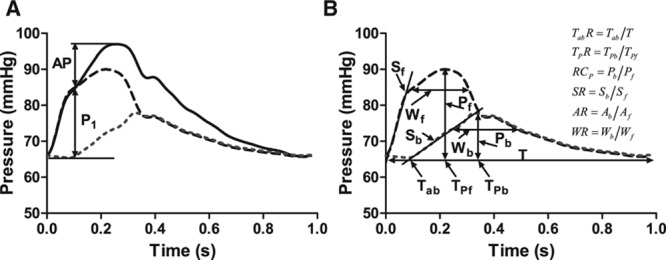
**A**, Central aortic pressure waveform showing the height above diastolic pressure of the first systolic shoulder (P1) and augmentation pressure (AP). Also shown are the forward (dashed line) and backward (dotted line) waves that summate to equal total pressure (solid line). **B**, Definition of wave morphology. *T*_ab_*R*, the time of arrival of the backward wave relative to the forward wave was measured as a proportion of the total period of the pressure pulse (*T*). Dimensionless parameters were used to describe the morphology of the backward wave relative to that of the forward wave: *T*_P_*R*, the ratio of time (from the start of systole) to the peak of backward wave (*T*_Pb_) to that of the peak of forward wave (*T*_Pf_); RC_p_, the ratio of the peak value of backward wave (*P*_b_) to that of the forward wave (*P*_f_); SR, the ratio of the maximum slope of the upstroke of the backward wave (*S*_b_, measured over the range 20%–80% of its peak value) to that of the forward wave (*S*_f_); WR, the ratio of width at 80% of peak value of the backward wave (*W*_b_) to that of the forward wave (*W*_f_); and AR, the ratio of area under backward wave (*A*_b_) to the area under forward wave (*A*_f_). AR indicates area ratio.


Ensemble averaged carotid pressure was used as surrogate for ascending aortic pressure.^10^ This together with aortic flow velocity was processed offline using custom software in MATLAB (MathWorks, Natick, MA) for wave separation analysis. The first systolic shoulder/peak (P1) of the aortic pressure waveform was identified as the first local minimum of the first derivative of the pressure curve (and confirmed by visual inspection by an observer blinded to the results) to determine the pulsatile component of BP at P1 (Figure 1) and augmentation pressure (AP, the difference between pressure at the second systolic peak, P2, and that at the first shoulder/peak). Wave decomposition was based on the conservation of mass and momentum and performed using Parker’s time-domain approach^2^ to obtain forward (*P*_f_) and backward (*P*_b_) pressure components of central pulse pressure so that *P*_f_+*P*_b_=*P*−*P*_d_, where *P* is total pressure and *P*_d_ is the diastolic pressure. *P*_f_ and *P*_b_ are given by

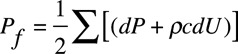
(1)


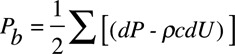
(2)

where *U* is flow velocity, ρ is blood density, and *c* is PWV, which was calculated using the method of the sum of squares.^11^

### Waveform Timing and Morphology

The time of arrival of the backward wave (*T*_ab_*R*) relative to the forward wave was measured as a proportion of the total period of the pressure pulse (*T*). Dimensionless parameters were used to describe the morphology of the backward wave relative to that of the forward wave (Figure 1): peak time ratio (*T*_P_*R*), the ratio of time from the start of systole to the peak of backward wave (*T*_Pb_) to that of the peak of forward wave (*T*_Pf_); reflection coefficient for peak values (amplitudes) of forward and backward waves (RC_p_), the ratio of the peak value of the backward wave (*P*_b_) to that of the forward wave (*P*_f_); peak slope ratio, the ratio of the maximum slope of the upstroke of the backward wave (*S*_b_, measured over the range 20%–80% of its peak value) to that of the forward wave (*S*_f_); area ratio, the ratio of area under backward wave (*A*_b_) to the area under forward wave (*A*_f_); and width ratio, the ratio of width at 80% of peak value of the backward wave (*W*_b_) to that of the forward wave (*W*_f_). A graphical comparison of relative waveform morphology between groups and effects of drugs on relative waveform morphology was made by calculating a scaling factor at each point in the cardiac cycle for height of the backward wave relative to the forward wave in one group or intervention (drug versus control) and then applying this to the other group or intervention.

### Numeric Modeling

A previously described 55-segment model that, for a given prescribed aortic flow, generates physiological aortic pulse waveforms^12^ was used for numeric simulations. Each artery of the network is characterized by its diameter, length, wall thickness, and arterial wall stiffness. The peripheral branches of the model are coupled to 3-element Windkessel models that represent the resistive and capacitive effects of the distal vessels. For the purpose of this study, a virtual database of 3325 healthy adult subjects with cardiac and arterial parameters spanning the physiological range^13^ was used to study the relationship between forward and backward waves. The parameters varied included heart rate, stroke volume, the stiffness, and diameter of elastic and muscular conduit arteries and peripheral resistances. Because some combinations of these parameters result in nonphysiological features of the aortic pulse, combinations were restricted to those that generated a BP (at the brachial artery) with diastolic pressure >40 mm Hg, systolic pressure <200 mm Hg, and pulse pressure >25 and <100 mm Hg.

### Statistics

A sample size of >150 was calculated to give >80% power (*P*<0.05) to detect a difference in reflection coefficient of >0.06 (≈20% of a typical value of 0.3) for subjects in the lowest and highest tertiles of pulse pressure. Subject characteristics are presented as means±SD, and results are as means±SE. Comparison of subject characteristics across tertiles of pulse pressure was made by one-way analysis of variance or (for categorical variables) by χ^2^ test. Differences in hemodynamic characteristics across tertiles of pulse pressure were also sought using one-way analysis of variance. Effects of drugs were examined using analysis of variance for repeated measures. Analysis was performed using SPSS version 19 and *P* value <0.05 was taken as significant. Ninety-five percent confidence intervals were calculated for key negative results.

## Results

### Central Hemodynamics in Patients With Hypertension

Compared with subjects in group 1, subjects in group 3 were characterized by increased central aortic pulsatility with mean values of P1, P2, and AP greater than those in group 1 by 21.0±2.5, 32.8±3.1, and 11.8±1.6 mm Hg, respectively. P1 and P2 were of approximately equal magnitude in group 1, so that AP was close to zero. However, in groups 2 and 3, P2 exceeded P1 with an AP of 11.0±1.6 mm Hg in group 3, and in most subjects, central pulse pressure was equal to P2.

**Figure 2.**
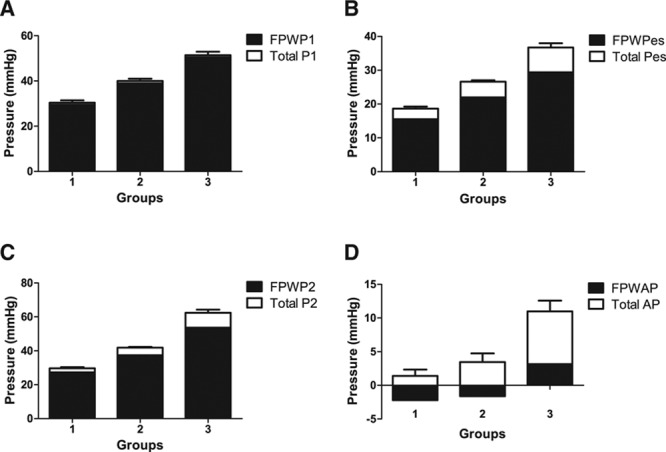
Pressure pulse components showing total pressure pulse and forward (FPW) component of pressure pulse for (**A**) first systolic peak (P1); (**B**) end-systolic pressure (*P*_es_); (**C**) second systolic peak (P2); and (**D**) augmentation pressure (AP).

Wave separation analysis demonstrated that, in group 1, both P1 and P2 were determined mainly by the forward wave, although, in subjects with a positive AP, the backward wave did contribute to P2 (Figure 2A and 2C). In group 3 (in which AP was positive, Figure 2D), P1 was also determined by the forward component of the pressure wave, and the forward wave provided the major contribution to P2. However, the backward wave provided a greater contribution to P2 than in group 1 (8.73±1.05 compared with 2.34±0.68 mm Hg in group 1, *P*<0.01; Figure 2) and contributed 7.84±1.15 mm Hg to the total AP of 11.0±1.60 mm Hg in group 3 (Figure 2D). Thus, the backward wave provided a slightly greater contribution to central pulse pressure in group 3 compared with group 1: 12.9±1.4% versus 7.49±1.3%; Figure 2C). The contribution of the backward wave relative to the forward wave increased over the cardiac cycle, so that its contribution to end-systolic pressure (*P*_es_) was greater than to P1 or P2 (Figure 2B).

**Figure 3.**
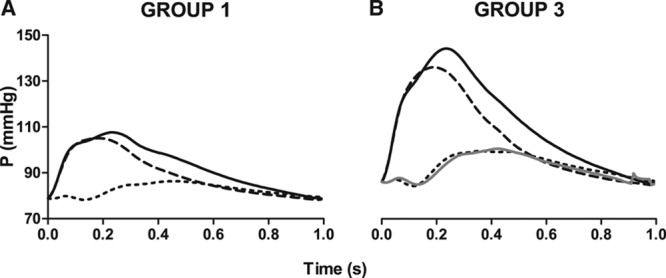
Average plots of pressure components in (**A**) group 1 and (**B**) group 3, showing measured pressure (solid line), forward pressure (dashed line), and backward pressure (dotted line). Grey solid line shows the backward wave calculated using the ratio of the backward to forward wave (at each point in the cardiac cycle) in group 1 (ie, assuming a constant backward/forward ratio at each point in the cardiac cycle).

The backward wave arrived earlier in subjects in group 3 compared with those in group 1 (95±0.4 versus 109±5.4 ms, *P*<0.05). However, the morphology of the backward wave relative to the forward wave was similar across the pulse pressure groups with no significant difference between RC_p_ and the ratios, slope ratio, width ratio, and area ratio, relating to the relative upslope, width at 80% of peak height, and areas across the pulse pressure groups (Table S1 in the online-only Data Supplement). The similarity of waveform morphology is depicted in Figure 3 in which the backward wave for group 3 is derived by applying a scaling factor to the forward wave in group 3 that is derived from the relative height of the backward to forward waves in group 1 (ie, a reflection coefficient calculated at each point in the cardiac cycle rather than from the peaks of backward and forward waves). The backward wave derived from the group 1 scaling factor is close to identical to that of the observed backward wave in group 3. The mean difference of reflection coefficient between these groups (mean for group 1−mean for group 3) was 0.004 with 95% confidence intervals of −0.040 to +0.049.

### Effects of Dobutamine, Norepinephrine, Phentolamine, and Nitroglycerin, on Central Hemodynamics in Normotensive Volunteers

**Figure 4.**
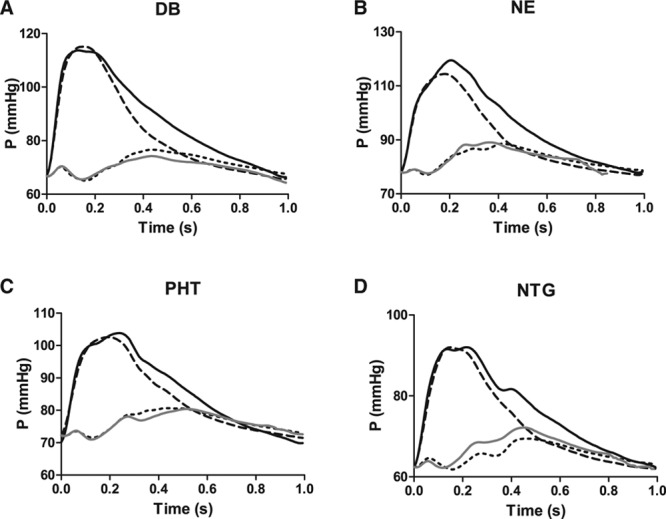
Measured pressure wave (black solid line) and decomposed forward (black dashed line) and backward (black dotted line) waves of the highest dose of (**A**) dobutamine (DB); (**B**) norepinephrine (NE); (**C**) phentolamine (PHT); and (**D**) nitroglycerin (NTG). The grey line is the backward wave calculated by applying the backward/forward ratio at baseline (at each point during the cardiac cycle) to the forward wave during drug infusion (ie, by assuming a constant backward/forward ratio at each point in the cardiac cycle).

Hemodynamic changes during inotropic/vasopressor and vasodilator stimulation are summarized in the Table S2. Changes in pulsatile and mean components of BP were as expected from the pharmacology of the drugs. Dobutamine with inotropic and vasodilator properties increased pulsatility (P1 and P2) but had no significant effect on diastolic BP. Norepinephrine increased diastolic BP and P2. Phentolamine decreased diastolic BP but had no significant effect on pulsatility. Nitroglycerin (NTG) decreased diastolic BP and P2. Dobutamine increased PWV, but the other drugs did not significantly influence PWV. RC_p_ decreased from 0.26±0.018 to 0.19±0.019 (*P*<0.01) during infusion of nitroglycerin. However, for all of the other drugs, RC_p_ remained similar at baseline and during drug infusion (mean differences [95% confidence interval] from baseline: 0.02 [−0.018 to 0.058], 0.01 [−0.047 to 0.067], and −0.001 [−0.035 to 0.033] for dobutamine, norepinephrine, and phentolamine, respectively. All other waveform morphology parameters also remained similar at baseline and during drug infusion (Figure 4; Table S3).

### Numeric Modeling

**Figure 5.**
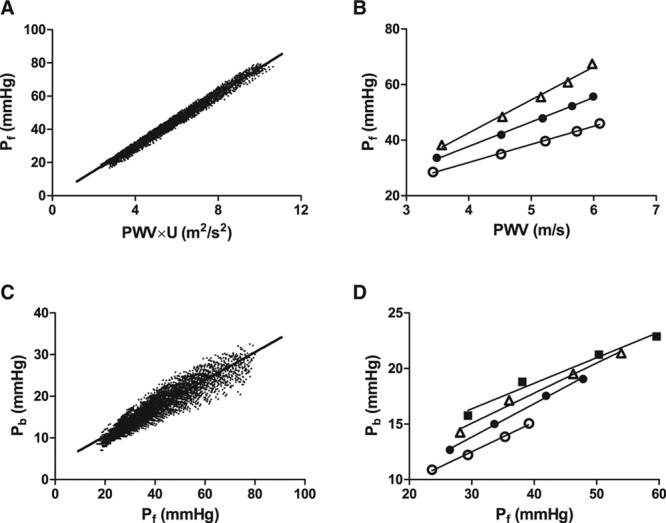
The relation of forward pressure wave amplitude (*P*_f_) to pulse wave velocity (PWV) and the relation of backward pressure wave amplitude (*P*_b_) to *P*_f_ as predicted by a 55-element branched arterial model. **A**, Relation between *P*_f_ and the product of PWV and aortic flow velocity (*U*) for the whole population. **B**, Relation between *P*_f_ and PWV when stroke volume (and hence *U*) is varied in discrete steps: −20% (○), 0% (● ), and +20% (Δ) of the median and other arterial properties held constant. **C**, Relation between *P*_b_ and *P*_f_ for the whole virtual population of 3325 subjects with arterial properties spanning the physiological range. **D**, Relation between *P*_b_ and *P*_f_ for the same range of values of large artery elastic modulus as in **C**, but variation of elastic modulus of muscular arteries is varied in discrete steps: −20% (○), 0% (● ), +15% (Δ), and +30% () of the median and other arterial properties held constant. This demonstrates that the scatter around the regression line in **C** stems mainly from variation in elastic modulus of muscular arteries.

Amplitude of the forward wave (*P*_f_) was mainly determined by the product of proximal aortic PWV and aortic flow velocity (Figure 5A; *R*=0.992, *P*<0.001 for the relation between forward wave amplitude and the PWV and aortic flow velocity product) with PWV and flow velocity accounting for approximately equal amounts of variance in *P*_f_ (Figure 5B). For the whole virtual database, the amplitude of the backward wave (*P*_b_) bore an approximately constant relationship to that of the forward wave (Figure 5C; *R*=0.931, *P*<0.001). However, when the compliance of muscular conduit arteries was varied independently from that of the aorta, the amplitude of the backward wave was seen to vary independently from that of the forward wave (Figure 5D), accounting for the relatively small amount of scatter around the regression line relating backward to forward wave amplitude in the whole virtual database.

## Discussion

### Contribution of Reflection and Backward Wave Morphology to Pulse Pressure in Hypertension

A major finding of this study is that, for different levels of pulse pressure, morphology of the backward pressure wave bears a constant relationship to that of the forward wave. Thus, a reflection coefficient defined as the ratio of the maximum height (amplitude) of the backward pressure wave to that of the forward wave or of the relative heights of each wave at any point throughout the cardiac cycle is constant across tertiles of pulse pressure. Although the backward wave is greater in those with high pulse pressure compared with low pulse pressure, its contribution to total pressure early in systole is relatively modest and, at any point in the cardiac cycle, it is proportionate to that of the forward wave. Thus, the forward wave is the dominant determinant of pulse pressure, and the amount of reflection as measured from the ratio of backward to forward wave does not play a major role in contributing to a raised pulse pressure in hypertension. Earlier arrival of the backward wave was observed in subjects with greater pulse pressure, but this led to only a minor increase in contribution of the backward wave relative to the forward wave.

### Contribution of Reflection and Backward Wave Morphology to Change in Pulse Pressure Generated by Vasoactive Drugs

We also observed similar morphology of the backward relative to the forward wave when comparing drugs that act mainly to increase myocardial contractility, constrict peripheral resistance vessels (norepinephrine), and dilate peripheral resistance vessels (phentolamine). Thus, despite wide perturbation of ventricular dynamics and peripheral resistance, reflection coefficient remained constant. The predominant action of these drugs is to increase myocardial contractility with some peripheral vasodilation (dobutamine), constrict peripheral resistance vessels with some increase in myocardial contractility (norepinephrine), and dilate peripheral resistance vessels (phentolamine). The exception to this was NTG, which has a specific action to dilate muscular conduit arteries.^14^ NTG produced a significant reduction in reflection so that the backward wave was reduced relative to the forward wave. This occurred in the absence of any change in PWV and confirms that selective modulation of muscular conduit vessel tone can influence the amount of reflection as previously suggested.^15,16^

### Comparison of Experimental Results With Numeric Modeling Approach

Although the theory of wave separation invokes few assumptions, being dependent on basic physical principles of conservation of mass and momentum, it is subject to experimental error in determining aortic flow velocity and estimating aortic pressure from carotid tonometry. A complimentary approach, therefore, is to use a numeric simulation.^12^ This removes experimental error but is dependent on the accuracy of the numeric model and parameters assigned to the elements of the model. Using a realistic model and with a wide range of parameters spanning the physiological range,^13^ we obtained results that were entirely consistent with the experimental results. Thus, the amplitude of the forward wave was determined by ventricular dynamics and aortic PWV, and the backward wave was proportional to the forward wave but with some variance. This variance of the amplitude of the backward wave relative to that of the forward wave (ie, variance in reflection) was explained by variation in the compliance of muscular conduit arteries, which would be compatible with the observed experimental influence of NTG on reflection. Taken together, our experimental results in patients with hypertension, in normotensive subjects under the influence of inotropic, vasopressor and vasodilator drugs, and theoretical numeric simulations, strongly support previous findings that the forward wave is the dominant determinant of pulsatile components of BP early in the systole.^8^

### Limitations

We studied predominantly middle-aged subjects with hypertension, who were on treatment, and it is possible that, within the general population, the contribution of reflection to the age-related increase in pulse pressure is more important than in essential hypertension; our conclusions may not be valid in untreated subjects. In a large community-based study, Torjesen et al^17^ have shown amount of reflection as assessed by reflection coefficient to be an important determinant of AP which, in turn, contributes a relatively large proportion of the age-related increase in pulse pressure, especially in middle-aged women.^18^ Even within the study of Torjesen et al,^17^ however, there was much greater variation of the forward wave compared with that of the backward wave over the lifespan, with forward wave amplitude varying, on average, by ≈30 mm Hg from the third to ninth decade and with reflection approximately constant to within 15%.

### Perspectives

The characteristics of ventricular-vascular coupling, particularly pressure wave reflection and the contribution of the backward pressure wave to BP, are controversial. Previous studies have assessed reflection indirectly assuming AP or index to be a measure of reflection or have quantified reflection using a single measure of the ratio of the peak backward to forward wave. Here, we measure the ratio of backward to forward pressure over the whole of the cardiac cycle and find it to be largely invariant of pulse pressure or of modulation of cardiovascular function with inotropic, vasopressor or vasodilator drugs. These observations together with studies showing the dominance of the forward wave during exercise^19^ underline the importance of ventricular dynamics and proximal aortic PWV as the major determinants of physiological and pathophysiological variation in pulse pressure. Reflection can be modulated by NTG, which has a specific action to dilate muscular conduit arteries; although increased muscular conduit artery tone is unlikely to contribute significantly to pulse pressure in hypertension, organic nitrates may be an effective treatment for reducing central pulse pressure both through a reduction in reflection and through a direct action on ventricular dynamics.^20^

### Conclusions

The ratio of backward to forward central aortic pressure remains approximately constant across groups with differing pulse pressure. Therefore, increased reflection is unlikely to contribute to increased pulse pressure in hypertension. However, the amount of reflection can be reduced by selective dilation of muscular arteries.

## Sources of Funding

This research was supported by the British Heart Foundation (RE/13/2/30182) and EPSRC (EP/K031546/1). We acknowledge financial support from the Department of Health via the National Institute for Health Research (NIHR) comprehensive Biomedical Research Centre and Clinical Research Facilities awards to Guy’s and St Thomas’ NHS Foundation Trust in partnership with King’s College London and King’s College Hospital NHS Foundation Trust.

## Disclosures

P. Chowienczyk and King’s College London have a financial interest in Centron Diagnostics. The other authors report no conflicts.

## Supplementary Material

## Novelty and Significance

### What Is New?

- This is the first study to examine the ratio of backward to forward central pressure throughout the cardiac cycle.

### What Is Relevant?

- Our finding of a constant ratio of backward to forward pressure across hypertensive groups with a 2-fold difference in pulse pressure and in normotensive subjects in the presence of inotropic, vasopressor and vasodilator stimulation suggests that variation in the amount of reflection contributes little to that of pulsatile components of blood pressure. However, reflection can be reduced by selective dilation of muscular arteries, and this may be an effective treatment to reduce central pulse pressure.

### Summary

Increased reflection as measured by backward/forward wave ratio contributes little to raised pulse pressure in hypertension but can be modified by dilation of peripheral muscular arteries.
