# Supplementary material for: Forward and Backward Pressure Waveform Morphology in Hypertension
Source: Hypertension. 2017 Jan 11;69(2):375–81. doi: 10.1161/HYPERTENSIONAHA.116.08089 (PMC5222553; doi:10.1161/HYPERTENSIONAHA.116.08089)
Supplement: Supplementary file 1 [file hyp-69-375-s001.docx]

**Forward and backward pressure waveform morphology in hypertension**

**Data Supplement**

Ye Li^1^

Haotian Gu^1^

Henry Fok^1^

Jordi Alastruey^2^

Phil Chowienczyk^1^

^1^King’s College London, British Heart Foundation Centre

^2^King’s College London, Division of Imaging Sciences and Biomedical Engineering

**Correspondence**

Prof PJ Chowienczyk, Department of Clinical Pharmacology, St Thomas’ Hospital, Lambeth Palace Road, London SE1 7EH, UK

Tel: + 44 207 1884642, Fax: + 44 207 4012242, E-mail: [phil.chowienczyk@kcl.ac.uk](mailto:phil.chowienczyk@kcl.ac.uk)

**Table S1 Wave morphology in hypertensive subjects by pulse pressure tertiles**

| PP tertile | T_ab_R | T_P_R | RC_p_ | SR | WR | AR |
| --- | --- | --- | --- | --- | --- | --- |
| 1 | 0.12±0.005 | 2.81±0.13 | 0.29±0.013 | 0.25±0.012 | 0.82±0.045 | 0.30±0.023 |
| 2 | 0.11±0.005 | 2.71±0.13 | 0.29±0.015 | 0.25±0.011 | 0.76±0.042 | 0.33±0.038 |
| 3 | 0.10±0.003 | 2.51±0.13 | 0.29±0.014 | 0.23±0.011 | 0.81±0.057 | 0.33±0.026 |
| P value | 0.018 | 0.25 | 0.94 | 0.62 | 0.69 | 0.81 |

PP, pulse pressure; T_ab_R, the time of arrival of the backward wave relative to the forward wave as a proportion of the total period of the pressure pulse; T_P_R, the ratio of time (from the start of systole) to the peak of backward wave to that of the peak of forward wave; RC_p_, the ratio of the peak value of backward wave to that of the forward wave; SR, the ratio of the maximum slope of the upstroke of the backward wave to that of the forward wave; WR, the ratio of width at 80% of peak value of the backward wave to that of the forward wave; AR, the ratio of area under backward wave to the area under forward wave.

**Table S2 Haemodynamic changes during infusion of inotropic, vasopressor and vasodilator drugs**

| Drug | HR (bpm) | DBP (mmHg) | P1 (mmHg) | P2 (mmHg) | PWV (m/s) | U_max_ (m/s) |
| --- | --- | --- | --- | --- | --- | --- |
| Dobutamine (μg/kg/min) | | | | | | |
| baseline | 64.7±3.51 | 65.4±3.10 | 36.8±3.74 | 32.5±3.02 | 4.07±0.40 | 1.16±0.067 |
| 2.5 | 66.7±4.51 | 64.7±2.80 | 43.7±3.23 | 38.0±2.35 | 4.52±0.37 | 1.32±0.049 |
| 5 | 69.0±4.74 | 66.8±2.30 | 51.3±3.76 | 41.4±2.47 | 5.11±0.39 | 1.37±0.041 |
| 7.5 | 73.0±5.24 | 66.8±2.02 | 59.0±3.41 | 47.2±3.17 | 5.59±0.32 | 1.42±0.044 |
| P value | 0.024 | 0.58 | <0.001 | 0.002 | 0.003 | 0.007 |
| Norepinephrine (ng/kg/min) | | | | | | |
| baseline | 60.9±3.20 | 66.2±3.05 | 35.5±1.67 | 33.0±2.67 | 4.35±0.29 | 1.14±0.032 |
| 12.5 | 56.5±3.17 | 71.1±3.03 | 36.5±2.05 | 35.8±3.18 | 4.71±0.40 | 1.15±0.038 |
| 25 | 54.3±3.11 | 74.2±3.00 | 33.1±1.43 | 37.3±2.72 | 4.69±0.30 | 1.07±0.054 |
| 50 | 52.3±2.93 | 78.7±2.93 | 36.8±2.84 | 43.7±4.31 | 5.56±0.60 | 1.07±0.056 |
| P value | <0.001 | <0.001 | 0.25 | 0.001 | 0.11 | 0.24 |
| Phentolamine (μg/min)* | | | | | | |
| baseline | 61.9±1.73 | 75.7±3.02 | 30.4±1.80 | 31.8±3.14 | 4.40±0.39 | 1.03±0.042 |
| 25 | 61.3±2.36 | 72.2±3.53 | 32.1±3.00 | 34.0±3.48 | 4.50±0.42 | 1.11±0.043 |
| 50 | 62.8±2.72 | 71.0±4.12 | 32.8±1.98 | 32.5±2.77 | 4.10±0.35 | 1.22±0.045 |
| 100 | 62.6±2.31 | 70.2±3.58 | 36.1±3.62 | 33.7±4.14 | 4.78±0.40 | 1.16±0.065 |
| P value | 0.39 | 0.021 | 0.13 | 0.42 | 0.41 | 0.098 |
| Nitroglycerine (μg/min) | | | | | | |
| baseline | 63.4±2.65 | 69.4±3.43 | 36.6±2.95 | 36.0±3.85 | 5.19±0.57 | 1.09±0.057 |
| 3 | 59.8±2.84 | 64.9±3.29 | 34.8±2.16 | 32.4±4.29 | 4.82±0.26 | 1.10±0.044 |
| 10 | 61.2±2.49 | 65.0±3.30 | 35.1±2.42 | 30.4±4.22 | 5.10±0.45 | 1.08±0.048 |
| 30 | 62.1±2.49 | 62.5±3.91 | 35.8±2.70 | 29.6±4.95 | 5.19±0.42 | 1.07±0.031 |
| P value | 0.26 | 0.018 | 0.70 | 0.033 | 0.63 | 0.79 |

HR, heart rate; DBP, diastolic blood pressure; P1, height above DBP of the first systolic shoulder of aortic pressure; P2, height above DBP of the second systolic shoulder of aortic pressure; PWV, pulse wave velocity; U_max_, maximum flow velocity. *With preceding boluses of 1, 2 and 4 mg for infusions of 25, 50 and 100 μg/min respectively.

**Table S3 Effects of drugs on waveform morphology**

| Drug | T_ab_R | T_P_R | RC_p_ | SR | WR | AR |
| --- | --- | --- | --- | --- | --- | --- |
| Dobutamine (μg/kg/min) | | | | | | |
| baseline | 0.12±0.013 | 3.30±0.30 | 0.26±0.018 | 0.20±0.019 | 0.99±0.18 | 0.29±0.036 |
| 2.5 | 0.12±0.016 | 3.79±0.42 | 0.28±0.023 | 0.18±0.017 | 0.80±0.11 | 0.31±0.034 |
| 5 | 0.12±0.018 | 4.03±0.29 | 0.25±0.020 | 0.18±0.011 | 0.90±0.10 | 0.30±0.049 |
| 7.5 | 0.10±0.022 | 3.87±0.34 | 0.25±0.015 | 0.21±0.017 | 0.98±0.14 | 0.34±0.051 |
| P value | 0.89 | 0.16 | 0.44 | 0.44 | 0.37 | 0.67 |
| Norepinephrine (ng/kg/min) | | | | | | |
| baseline | 0.12±0.011 | 3.05±0.41 | 0.26±0.025 | 0.20±0.024 | 0.84±0.14 | 0.28±0.049 |
| 12.5 | 0.12±0.010 | 2.65±0.31 | 0.28±0.017 | 0.23±0.027 | 0.80±0.13 | 0.32±0.029 |
| 25 | 0.11±0.012 | 2.61±0.21 | 0.29±0.016 | 0.29±0.034 | 0.83±0.081 | 0.33±0.022 |
| 50 | 0.09±0.006 | 2.46±0.035 | 0.25±0.011 | 0.25±0.025 | 0.71±0.10 | 0.26±0.037 |
| P value | 0.072 | 0.32 | 0.32 | 0.33 | 0.57 | 0.40 |
| Phentolamine (μg/min)* | | | | | | |
| baseline | 0.12±0.006 | 3.08±0.22 | 0.23±0.017 | 0.23±0.017 | 0.88±0.15 | 0.24±0.038 |
| 25 | 0.12±0.007 | 3.15±0.30 | 0.25±0.026 | 0.19±0.021 | 0.81±0.092 | 0.28±0.041 |
| 50 | 0.12±0.008 | 3.16±0.23 | 0.27±0.027 | 0.24±0.022 | 0.84±0.13 | 0.28±0.037 |
| 100 | 0.13±0.119 | 3.09±0.38 | 0.23±0.019 | 0.19±0.018 | 1.06±0.21 | 0.26±0.034 |
| P value | 0.49 | 0.67 | 0.39 | 0.027 | 0.75 | 0.58 |
| Nitroglycerin (μg/min) | | | | | | |
| baseline | 0.11±0.006 | 3.04±0.32 | 0.26±0.018 | 0.21±0.018 | 0.78±0.055 | 0.27±0.037 |
| 3 | 0.12±0.011 | 3.14±0.31 | 0.23±0.012 | 0.24±0.024 | 1.12±0.11 | 0.25±0.037 |
| 10 | 0.13±0.012 | 3.14±0.44 | 0.21±0.022 | 0.24±0.031 | 0.90±0.20 | 0.19±0.044 |
| 30 | 0.12±0.008 | 2.88±0.49 | 0.19±0.019 | 0.26±0.031 | 1.52±0.31 | 0.22±0.059 |
| P value | 0.68 | 0.85 | 0.008 | 0.48 | 0.19 | 0.27 |

T_ab_R, the time of arrival of the backward wave relative to the forward wave as a proportion of the total period of the pressure pulse; T_P_R, the ratio of time (from the start of systole) to the peak of backward wave to that of the peak of forward wave; RC_p_, the ratio of the peak value of backward wave to that of the forward wave; SR, the ratio of the maximum slope of the upstroke of the backward wave to that of the forward wave; WR, the ratio of width at 80% of peak value of the backward wave to that of the forward wave; AR, the ratio of area under backward wave to the area under forward wave. *With preceding boluses of 1, 2 and 4 mg for infusions of 25, 50 and 100 μg/min respectively.
